# A Review of KSHV: Lifecycle, Tumorigenesis, Immune Evasion and Therapeutic Strategies

**DOI:** 10.3390/v18101053

**Published:** 2026-09-23

**Authors:** Chaoran Zhuo, Dongdong Cao, Fangling Li

**Affiliations:** School of Medicine, Shihezi University, Shihezi 832002, China; 15020979066@163.com

**Keywords:** Kaposi’s sarcoma-associated herpesvirus, RTA, immune evasion, therapeutic strategies

## Abstract

Kaposi’s sarcoma-associated herpesvirus (KSHV) is an important human gamma-herpesvirus, particularly with a significant incidence rate among AIDS patients and other immunosuppressed populations. This review introduces the basic virological characteristics of KSHV and elucidates the core molecular mechanisms underlying KSHV tumorigenesis. Clinical management primarily focuses on KSHV-related diseases, utilizing antiretroviral therapy, chemotherapy, radiotherapy, and therapies targeting specific targets. However, there still lack of specific anti-KSHV drugs in terms of treatment. Current research is increasingly directed toward novel therapeutic strategies, including those that sustain viral latency or promote lytic reactivation, alongside antigen-based immunotherapeutic approaches. Despite significant breakthroughs in research, the precise regulatory network of KSHV latent infection, the complex interactions between the virus and the host microenvironment (especially inflammatory and immune cells), as well as the development of highly effective and low-toxicity specific antiviral drugs, remain major challenges for the future. A deep understanding of the pathogenic mechanisms of KSHV will lay a solid foundation for the development of more effective prevention, diagnosis, and treatment methods.

## 1. Introduction

Kaposi’s sarcoma-associated herpesvirus (KSHV), also known as human herpesvirus 8 (HHV-8), belongs to the gamma-herpesvirus subfamily [1]. The Hungarian dermatologist Moritz Kaposi described multiple, pigmented sarcomas appearing on the skin of elderly men, mostly of Mediterranean or Eastern European Jewish descent in 1872 [2]; the disease was later named Kaposi’s sarcoma (KS). In 1994, Chang Y. at Columbia University in New York, USA, made a groundbreaking discovery using representational difference analysis (RDA)—a PCR-based technique: the DNA sequences of a new gamma-herpesvirus were consistently present in KS lesions but absent from unaffected skin and from most other diseased tissues, thus lending the name KSHV [3,4]. KSHV was first isolated in skin biopsies acquired from AIDS patients with KS. Currently, KSHV can be detected in all KS lesions [5], albeit at different percentages of infected cells, regardless of lesion origin or clinical subtype, and KSHV could cause a series of diseases, such as primary effusion lymphoma, multicentric Castleman’s disease (MCD), KSHV inflammatory cytokine syndrome (KICS), and could even cause increased risk of diabetes, malaria and other cancers [6].

KSHV infection has obvious regional and population differences; overall, sub Saharan Africa is a highly prevalent area, as well as regions such as the Mediterranean coast and Xinjiang in China, while most of North America, Northern Europe, and East Asia are low-prevalence areas [7,8,9]. A large amount of evidence has demonstrated that KS patients harbor KSHV, yet additional cofactors, including HIV infection, contribute to disease development [10,11]. Analysis of global KSHV samples has revealed multiple infection subtypes. KSHV has been classified into seven major molecular subtypes, subtypes A, A5, B, C, D, E and F, based on the K1 open reading frame (ORF) [12,13]. The specific characteristics of the first viral gene (ORF-K1) encodes a glycosylated transmembrane protein composed of 289 amino acids, a protein also found in the immunoglobulin receptor family [14]. Mutations in this gene are primarily located within the VR1 (54–93 AA) and VR2 (191–228 AA) domains [15,16]. Subtypes A and C are closely related and are generally considered to coexist, found predominantly in Western and Mediterranean countries, whereas subtypes A5 and B are concentrated in sub-Saharan Africa (SSA), where they represent the great majority of KSHV infections. In particular, subtype A5 is more prevalent in patients with AIDS-related Kaposi’s sarcoma and those presenting with multiple lesions [17]. Subtypes E and D originate from Native American regions and Pacific islands, including Japan [16]. The evolution and dynamics of KSHV infection partially reflect human migration history.

## 2. KSHV Genome and Lifecycle

KSHV exists in two distinct phases during its lifecycle as other herpesviruses, the default latent replication and lytic replication [18]. The KSHV only expresses limited proteins to evade the host cell immune response and promote virus survival in the latent state, whereas the viral genome replicates as linear DNA to facilitate massive viral replication in the lytic phase [19]. However, in order to avoid immune damage and maintain survival, KSHV mainly exists in the latent state, and will enter the lytic state under several physiological factors, including hypoxia, HIV infection, inflammatory cytokines, oxidative stress, and ROS [18]. In KSHV-infected PEL cell lines, the proportion of cells that spontaneously enter the lytic cycle is generally low, typically ranging from less than 1% to approximately 5% [20], while in a variety of cells successfully infected with KSHV in vitro, KSHV remains dormant. Under the stimulation of phorbol 13-acetate (12-O-Tetradecanoylphorbol 13-acetate, TPA) and sodium butyrate, the latent virus could be induced to enter the lytic state [21,22].

KSHV encodes different pathogenic proteins in the latent and lytic state (Figure 1A). The virus genes encoded in the latent state include latency-associated nuclear antigen (LANA) encoded by ORF73, FLICE inhibitory protei (vFLIP) encoded by ORF71, viral interferon regulatory factor (vIRF) encoded by ORF K9, and 25 mature microRNAs (miRNAs) [4,23]. The pathogenic proteins encoded by the lytic state include lytic cycle gene encoded by ORF26, vBCL-2 encoded by ORF16, vIL-6 encoded by ORFK2, transmembrane glycoprotein K1 encoded by ORFK1, and v-GPCR encoded by ORF74 [24,25]. Among them, the Replication and Transcription Activator (RTA) encoded by ORF50 is the molecular switch for the transition of KSHV from latent to lytic state [26].

By inducing the expression of lytic genes, RTA controls viral gene DNA replication and encodes viral structural protein recombination [27], and regulates and expresses host genes. RTA is composed of 691 amino acids [28,29], including multiple domains; the N-terminal is the DNA binding domain, the C-terminal is the trans-activation domain, in addition to the proline enrichment region, the basic amino acid enrichment region, the serine/threonine enrichment region, the cysteine/histidine enrichment region, the hydrophobic acid repetition region, and the leucine heptapeptide repetition domain. There are two nuclear localization signals (NLSs) and dimerization and tetramerization domains. RTA regulates cellular genes by directly binding RTA response element (RRE) sequence-specific DNA binding sites or by binding them to other transcription factors [30,31], and can transactivate LANA promoters [32]. LANA, the main regulator of KSHV latent state, is composed of 1021–1162 amino acids and divided into the N-terminal region, central region and C-terminal region [33]. The N-terminal region contains dominant nuclear localization signal and histone H2A/H2B binding region interacting with host chromosomes [34]. The C-terminal region has a DNA-binding domain and a dimerization domain that interact with the LANA binding site in the terminal repeat of the genome [35]. LANA directly or indirectly interacts with other factors through different signaling pathways to regulate cell proliferation, apoptosis and gene transcription. Furthermore, LANA binds to the hypoxia response element (HRE) within the RTA promoter to enhance RTA gene expression [15,36], facilitating the transition from latent to lytic replication (Figure 1B).

Moreover, KSHV encodes 12 precursor microRNAs that are processed into 25 mature microRNAs [15]. These 25 miRNAs regulate the viral cycle in two ways: one is direct action on the virus, such as direct interference or cleavage; for example, miR-K9-5p and miR-K7 inhibit RTA to help KSHV maintain latency [15,37]. The second mechanism involves targeting cellular genes; for instance, miR-K11 modulates B cells to promote KSHV latency, thereby facilitating tumorigenesis [38]. Concurrently, it disrupts host immune surveillance by suppressing NK cells and promoting inflammatory cytokine secretion [39], ultimately inducing KS development. KSHV miRNAs also regulate cellular metabolism in infected cells, making the manipulation and fine-tuning of metabolic pathways critical for KS cell proliferation and survival [40,41].

## 3. Latency and Lytic Switch and Signaling Pathways

Studies indicate that the lytic replication cycle directly promotes tumorigenesis by transmitting the virus to target cells and providing paracrine regulation for KS development [25,42]. Lytic replication plays a novel role in sustaining latently infected cell populations, which would otherwise rapidly lose their latent viral epigenome following mitotic division. This indicates that KSHV lytic replication and the continuous primary infection of fresh cells are critical for the virus oncogenic and pathogenic mechanisms. Notably, studies have documented instances of abortive lytic replication, in which certain lytic genes are expressed but no progeny virions are produced [43].

RTA plays a pivotal role in driving the transition from the latent to the lytic cycle. Once initiated, RTA forms a positive feedback loop by binding to its own promoter region, thereby amplifying its expression levels and establishing the essential conditions for lytic viral replication [44,45,46].

### 3.1. Notch Signaling Pathway

RBP-Jκ, as the primary transduction factor and mediator of Notch signaling, interacts with RTA and recruits it to the KSHV promoter, thereby inducing viral genes—a process critical for KSHV reactivation [45]. Activated NF-κB forms a complex with RBP-Jκ, the RTA cofactor, thereby competitively inhibiting KSHV lytic activity. NF-κB also suppresses KSHV reactivation by blocking RBP-Jκ binding to the viral promoter, thus preventing RTA recruitment [24,45]. The K15 cytoplasmic tail exhibits high dynamism and is susceptible to tyrosine phosphorylation, which activates multiple signaling pathways including NF-κB [46] (Figure 2).

### 3.2. ROS

Environmental stress signals such as hypoxia and reactive oxygen species (ROS) accumulation can directly trigger the KSHV lytic cycle by inducing specific transcription factors to bind to the RTA promoter [47,48]. Tumor cells often reside in an oxidative stress state characterized by excessive ROS accumulation, and high ROS concentrations can disrupt KSHV latent equilibrium by inducing virulent replication, ultimately leading to infected cell apoptosis [49]. However, a critical contradiction exists: KS tumor cells can stably maintain latent KSHV infection within an oxidative stress microenvironment; this suggests that KSHV-infected cells may continuously enhance their antioxidant defense capabilities to resist ROS-mediated lytic activation [50]. Previous studies have confirmed that loss of expression of the antioxidant protein transcription factor FoxO1 promotes KSHV lytic reactivation by elevating intracellular ROS levels. Conversely, KSHV can maintain its latent state by upregulating FoxO1 expression through its own encoded microRNAs and vFLIP, which activate the NF-κB signaling pathway [44,51].

### 3.3. Hypoxia

Under hypoxic conditions, hypoxia-inducible factor 1α (HIF1α) can directly bind to the RTA promoter region, significantly enhancing RTA transcription activity and driving the virus into the lytic phase [37,52,53]. Concurrently, KSHV has evolved a unique regulatory strategy to balance intracellular HIF1α levels: its encoded v-Cyclin downregulates HIF1α expression, thereby creating suitable conditions for viral DNA replication in hypoxic environments and preventing premature host cell death caused by excessive activation of the lytic program [54]. HIF-2α is the predominant isoform in endothelial cells, though both HIF-α proteins can be expressed at similar levels under certain conditions. Notably, HIF-2α is preferentially enriched in endothelial cells compared with other cell types [55]. KSHV employs multiple strategies to aberrantly activate and stabilize HIF-2α under normoxia, thereby creating a hypoxia-like state. By regulating HIF2α levels and its ER localization under normoxia, KSHV promotes translation of viral lytic mRNAs via the HIF2α-dependent eIF4E2 initiation complex [56].

### 3.4. RTA mRNA

The stability of RTA mRNA is further regulated by host cell epigenetic modifications, specifically through dynamic control of N6-methyladenosine (m6A) modifications [57]. The N6-adenosine methyltransferase 70 kDa subunit (METTL3), serving as the core catalytic molecule for m6A modification, deposits m6A marks onto RTA mRNA, while YTH N6-methyladenosine RNA-binding protein-2 (YTHDF2) and staphylococcal nuclease domain-containing protein-1 (SND1) function as m6A recognition proteins [37,44], participating in regulating the degradation or translation efficiency of labeled RTA mRNA. Given METTL3’s pivotal role in tumorigenesis and viral regulation, targeted intervention against METTL3 has emerged as a highly promising direction for cancer therapy [53].

### 3.5. LANA Gene

The KSHV-encoded LANA plays a dual regulatory role in the latent-to-lytic switch: On one hand, LANA directly upregulates HIF-1α mRNA transcription while binding to the HRE in the RTA promoter region, thereby promoting expression of the lytic key molecule RTA and driving the transition from latent to lytic replication [51]. On the other hand, LANA negatively regulates RTA expression via the hypoxia-sensitive chromatin remodeler KAP1 [58], thereby balancing the degree of viral lytic activation. In the transcriptional regulation of KSHV lytic genes containing HREs, RBP-Jκ and HIF-1α form a synergistic complex to jointly initiate expression of these genes. Conversely, KAP1 antagonizes the interaction between RBP-Jκ and HIF-1α, thereby inhibiting lytic gene activation mediated by this complex and participating in the fine-tuning of KSHV latent-lytic balance [24] (Figure 2A).

### 3.6. Sox Gene

Upon entering the lytic state, KSHV releases viral particle proteins that suppress host gene expression. KSHV Sox, encoded by ORF37, is a protein with endonuclease activity that accelerates cytoplasmic mRNA degradation and blocks its nuclear export [59]. SOX affects both host and viral transcripts, reducing viral protein expression and maintaining viral particle synthesis equilibrium. Another KSHV protein encoded by ORF10 forms a complex with the cellular export factor Rae1 and its co-encoded nucleoprotein NUP98. This complex directly acts on the cellular mRNA export pathway to suppress host gene expression. The ORF37 and ORF10 genes retain mRNA within the nucleus, functioning to block nuclear export and suppress host gene expression. ORF37 modulates host gene expression with ORF10 assistance to optimize viral replication. Rae1-NUP98 is particularly crucial for viral replication and serves as a potential viral target [60]. Concurrently, the SOX protein interacts with the absent in melanoma 2 (AIM2) protein, disrupting its binding to double-stranded DNA (dsDNA). This inhibits AIM2-dsDNA aggregation and the recruitment of apoptosis-related speck-like proteins containing CARD (ASC), thereby suppressing AIM2-dependent inflammasome activation and senescence, ultimately inhibiting cell death [59,60]. This entire regulatory network achieves the switching between KSHV latency maintenance and lytic activation within host cells through multi-molecular, multi-pathway interactions. These regulatory nodes also provide potential targets for the targeted treatment of KSHV-associated diseases (Figure 2B).

## 4. Immune Evasion

During latency, KSHV expresses only a small fraction of related genes, specifically a subset encoded within the KSHV Latency-Associated Region (KLAR), including LANA, vFLIP, vCyclin, and 12 microRNAs [61]. Upon entering the host cells, KSHV establishes immune evasion to escape the attack of the immune system [15]. KSHV infection is primarily recognized and eliminated by CD8^+^ T cells and NK cells, which form a complementary immune system, while CD4^+^ T cells provide immune responses. CD8^+^ T cells recognize viral antigens presented by Major Histocompatibility Complex Class I (MHC-I) molecules on the cell surface, subsequently secreting cytokines, releasing viral particles, or inducing apoptosis via the Fas-mediated pathway [39,62] (Figure 3A). However, this pathway relies on normal MHC-I expression, which becomes impaired when its presentation is disrupted. NK cells compensate for this immunological gap by specifically targeting and eliminating cells lacking MHC-I expression while releasing cytotoxic granules; their full activation requires binding of the major histocompatibility complex class I-associated molecule (MICA/B) to the NKG2D receptor on NK cell surfaces [63]. CD4^+^ T helper cells lack direct cytotoxic activity but function by recognizing exogenous antigens presented by MHC-II molecules on phagocyte surfaces [39]. K3 is a KSHV-encoded lytic gene that recognizes the immune molecule MIR1, promoting degradation of MHC-I, CD1d, CD31, and interferon-γR1, thereby impairing CD8^+^ T cell function [64]. KSHV K5 encodes a lytic gene that recognizes the immune regulator MIR2, promoting degradation of MHC-I, CD54, B7-2, CD1d, MICA, and MICB degradation, disrupting normal CD8^+^ T cell and NK cell function [39,64]. Beyond these synergistic effects, MIR2 possesses unique functions: it downregulates ICAM-1 and B7-2 expression, reducing T cell activation, and enhances vascular permeability. KSHV LANA binds to and prevents IRF4 activation of the CIITA promoter, resulting in reduced MHC-II expression to evade CD4^+^ T cell-mediated immunity [65]. KSHV RTA promotes MHC-II proteasomal degradation while enhancing expression of the MHC-II antagonist membrane-associated ring CH (MARCH8) [39]. RTA also ubiquitinates and degrades certain proteasomes, notably the SMC5/6 complex [23]. This complex concentrates KSHV chromatin, significantly inhibiting viral transcription. RTA antagonizes SMC5/6, partially preventing KSHV from releasing viral particles. Consequently, the virus evades detection by the host immune system and can establish lifelong latent infection.

KSHV utilizes the viral interleukin vIL-6 encoded by K2 to promote the transformation of infected monocytes into macrophages [66]. These infected macrophages protect the viral reservoir and impair immune stimulatory functions. Sustained stimulation leads to dysfunction of host macrophages. This mechanism enables KSHV to evade host immune surveillance and even establish lifelong infection (Figure 3B). vIL-6 suppresses the expression of C-X-C chemokine ligand 8 (CXCL8) induced by the proinflammatory cytokine IL-1β, thereby inhibiting immune cell recruitment and preventing B cell infiltration during neutrophil infiltration, suppressing innate immunity [39,66,67]. RTA activates vIL-6 through direct or indirect interactions with the vIL-6 promoter, triggering Notch signaling mediated by the recombinant signaling binding protein (RBP-Jκ) in the Ig kappa J region [68]. KSHV also encodes three CC-chemokine ligands (CCLs) that negatively regulate inflammation and inhibit the migration of CD8^+^ T cells [39,69].

KSHV can suppress the expression of the host genes. The host cell could detect the virus through the binding of pathogen-associated molecular patterns (PAMPs) to pattern recognition receptors (PRRs), and the receptors promote the production of interferons and the transcription of antiviral genes [70]. Upon KSHV infection, host cells can trigger type I, II, and III interferons, activating immune responses against the pathogen, which includes restricting viral replication by increasing MHC-1 expression, potentially leading to growth arrest or apoptosis. To counteract this mechanism, KSHV encodes four viral homologs of intracellular IRFs (vIRF1 to vIRF4) [70,71], all of which are hijacked from the host genome. These proteins target multiple host cell proteins, including the endogenous IRFs, to suppress the interferon response. VIFs are not the only KSHV proteins regulating the interferon response; ORF45 competitively binds to IκB kinase ε (IKKε) and Tbk1 [71], both alternative substrates for IRF7 phosphorylation, thereby inhibiting IRF7 phosphorylation and activation. RTA functions as an E3 ubiquitin ligase [23], promoting IRF7 degradation via the proteasome pathway. Furthermore, LANA and basic region leucine zipper (K-bZIP) protein of KSHV directly bind the interferon-β promoter, blocking the interferon response pathway by inhibiting its transcriptional activity [37,44]. Over the long evolutionary history, KSHV has also developed expression of host-homologous proteins to confuse the host immune system.

## 5. Therapeutic Strategies

The extensive regulation of KSHV on the host immune system provides multiple directions for clinical intervention research, but the current therapeutic means cannot eradicate KSHV, and the purpose of anti-KS therapy is to slow the progression of the disease and alleviate the symptoms of patients. In the course of treatment, the spread of the disease, the limitation or spread of the lesion and the degree of the evolution of the disease should be taken into account, among which attention should be paid to the recovery of the patient’s immune status and the manifestation of complications. Because the clinical characteristics of different types of KS are different, the treatment should be selected individually. For patients with typical KS, local physical therapy is often used in mild cases, and low-dose interferon immunotherapy is often recommended when the disease is more severe, or if oral and visceral involvement and systemic symptoms occur [72,73]. In most patients with AIDS-associated KS, treatment with antiretroviral drugs to induce immune reestablishment is recommended early in the disease, followed by combination cytokine therapy depending on the severity of the disease [74,75].

For patients with refractory, disseminated and recurrent KS, multiple clinical trials have shown that the addition of anti-angiogenic immunomodulators and immune checkpoint inhibitors to regulate immunity has a certain therapeutic effect [76], and the immunotherapy mainly targets various immune receptor antibodies to restore or enhance immune function. One of the most effective immunotherapy approaches currently used in clinical practice is anti-programmed death receptor-1 (PD-1)/programmed death-ligand 1 (PD-L1) targeted therapy. PD-1, a key immunoregulatory molecule expressed on the surface of T cells, primarily functions to negatively regulate the immune effector functions of T cells [77,78]. PD-L1, as the ligand for PD-1, exists abundantly in both tumors and tumor-infiltrated T cells, B cells, macrophages, and DCs [79]. By specifically binding to PD-1 on T cell surfaces, it significantly suppresses T cell recognition and killing activity against tumor or infected cells, ultimately mediating tumor cell escape [80]. Notably, PD-L1 expression is also markedly upregulated in KSHV-infected monocytes [39,81]. This abnormal expression directly drives the formation of an immune evasion phenotype, creating favorable conditions for KSHV’s sustained survival and replication within host cells, thereby inducing a series of KSHV-associated diseases such as KS [82]. Based on these mechanisms, PD-1/PD-L1-targeted therapeutic strategies have been developed and applied clinically [83]. Future therapeutic approaches may harness controlled local hypoxia to activate the HIF1α pathway [84], triggering KSHV in latently infected cells to enter the lytic phase. Once KSHV lysis commences, infected cells express abundant viral lytic proteins such as RTA and ORF55, which serve as immune markers, making cells more readily recognized by T cells.

Nanomaterials have been used for small-molecule drug delivery against KSHV infection to overcome the drawbacks of poor water solubility and easy degradation. Organic nanoparticles represent the most extensively studied nanoparticle type in drug delivery research and the most widely approved system for human therapeutics [85]. Recently, zeolite imidazolate framework-8 (ZIF-8)—a biocompatible porous material composed of coordinated zinc ions and 2-methylimidazole—has been utilized to construct a co-loaded dual-drug nanocomposite (Ganciclovir (GCV) and miR-34a-5p) [86,87], with GCV encapsulated internally and miR-34a-5p adsorbed externally. A microneedle-structured nano-delivery system was further constructed using a polydimethylsiloxane (PDMS) microneedle mold to fabricate MN/GCV@ZIF-8 arrays [87,88], which not only demonstrated excellent transdermal penetration capability but also significantly suppressed viral cleavage and replication in vivo, exhibiting anti-KSHV functionality.

Simultaneously, a novel vector emerged: adeno-associated virus (AAV), a non-enveloped, single-stranded DNA virus with a genome size of approximately 4.8 kb [89,90]. By ingeniously leveraging LANA’s ability to maintain the stability of the terminal repeat (TR) circular genome and TR enhancement function for KSHV-induced promoters, researchers successfully developed a vector (AAV8-TR2-Orip-TK/GCV) capable of specifically expressing engineered thymidine kinase (TK) in KSHV-infected cells [91]. This was achieved by embedding two copies of the TR sequence carrying the lytic induction gene promoter (TR2-orip) into the TR sequence, which successfully developed a vector (AAV8-TR2-Orip-TK/GCV) capable of specifically expressing engineered TK in KSHV-infected cells. TK, encoded by ORF21, is exclusively expressed in γ-herpesviruses; it activates antiviral drugs during KSHV production by phosphorylating nucleoside analogs [92]. When combined with the anti-herpesvirus drug GCV, the vector efficiently eradicates various KSHV-infected cells, including induced pluripotent stem cell-derived epithelial-forming cells, without harming normal cells. Concurrently, the vector inhibits viral particle release from activated KSHV-infected cells. Notably, certain anticancer drugs known to activate KSHV can enhance TK expression within the vector, producing synergistic therapeutic effects when combined with the AAV8-TR2-Orip-TK/GCV regimen [91] (Figure 4).

Precision intervention strategies targeting viral protein functions have emerged as a novel therapeutic approach, focusing on key regulatory mechanisms governing KSHV lytic replication and infectious particle production [93,94]. Recent studies reveal that small-scale CRISPR knockout screening of viral genes indicates ORF55 knockout significantly suppresses progeny particle production [95,96]. Palmoylation of the KSHV-encoded ORF55 protein is essential for its localization to the Golgi apparatus, where it completes secondary viral envelope assembly and generates infectious progeny particles [95]. By screening for specific small-molecule compounds that block its Golgi localization, it is possible to inhibit KSHV lytic replication and infectious particle release at the source, thereby reducing viral transmission and the transition from latent to lytic states within the host. This may represent a novel strategy for suppressing lytic-phase viral DNA replication and hold promise for combination therapy with competitive DNA polymerase inhibitors in KS treatment.

Preventive vaccination helps the human body develop specific immunity against certain diseases; however, the KSHV virus possesses the ability to evade the host immune system, presenting significant challenges in developing such vaccines [97,98]. K8.1, gp, gB, and gH/gL glycoproteins are involved in the mechanism of viral invasion into host cells; those glycoproteins served as effective targets for neutralizing antibodies, thereby inducing an antiviral infection response [99,100,101]. Studies indicated that integrating KSHV gpK8.1, gB, and gH/gL glycoproteins onto viral particle surfaces effectively induces antibody production against KSHV [102,103], positioning those proteins as vaccine candidates. Researchers have developed a novel multivalent vaccine by integrating KSHV viral proteins K8.1, gp, gB, and gH/gL into a single multivalent KSHV-like particle (KSHV-LP) [104]; purified KSHV-LPs exhibit high similarity to natural KSHV viral particles in particle size, geometric morphology, and ultrastructure. Immunizing rabbits with KSHV-LPs combined with an adjuvant induced potent glycoprotein-specific humoral immune responses. Further purification and functional validation of immunoglobulins from immunized rabbits demonstrated their ability to effectively neutralize KSHV infection in epithelial, endothelial, fibroblast, and B-lymphocyte cell lines, achieving neutralization efficiencies of up to 60–90% within the tested concentration range [104]. These findings suggest that KSHV-LPs possess the antigenic epitope characteristics mimicking native KSHV and the ability to efficiently induce protective immune responses, potentially serving as an optimal candidate platform for developing safe and effective KSHV prophylactic vaccines.

## 6. Current Challenges and Future Perspectives

KSHV is a human tumor virus with an exceptionally complex pathogenic mechanism. By establishing latent infection and periodic lytic replication, it employs multiple viral factors—including various miRNAs and immunomodulatory proteins—to finely regulate host cell processes such as proliferation, survival, immune recognition, and metabolism. This enables tumorigenesis and immune evasion. Although current therapeutic strategies have achieved some success in controlling disease progression and alleviating symptoms, complete eradication of the virus remains a significant challenge. Future research should focus on elucidating the regulatory networks governing the KSHV life cycle and its interaction mechanisms with the host. This will lay a critical foundation for developing novel targeted therapies and preventive vaccines capable of effectively eliminating latent viral infections or preventing infection.

## Figures and Tables

**Figure 1:**
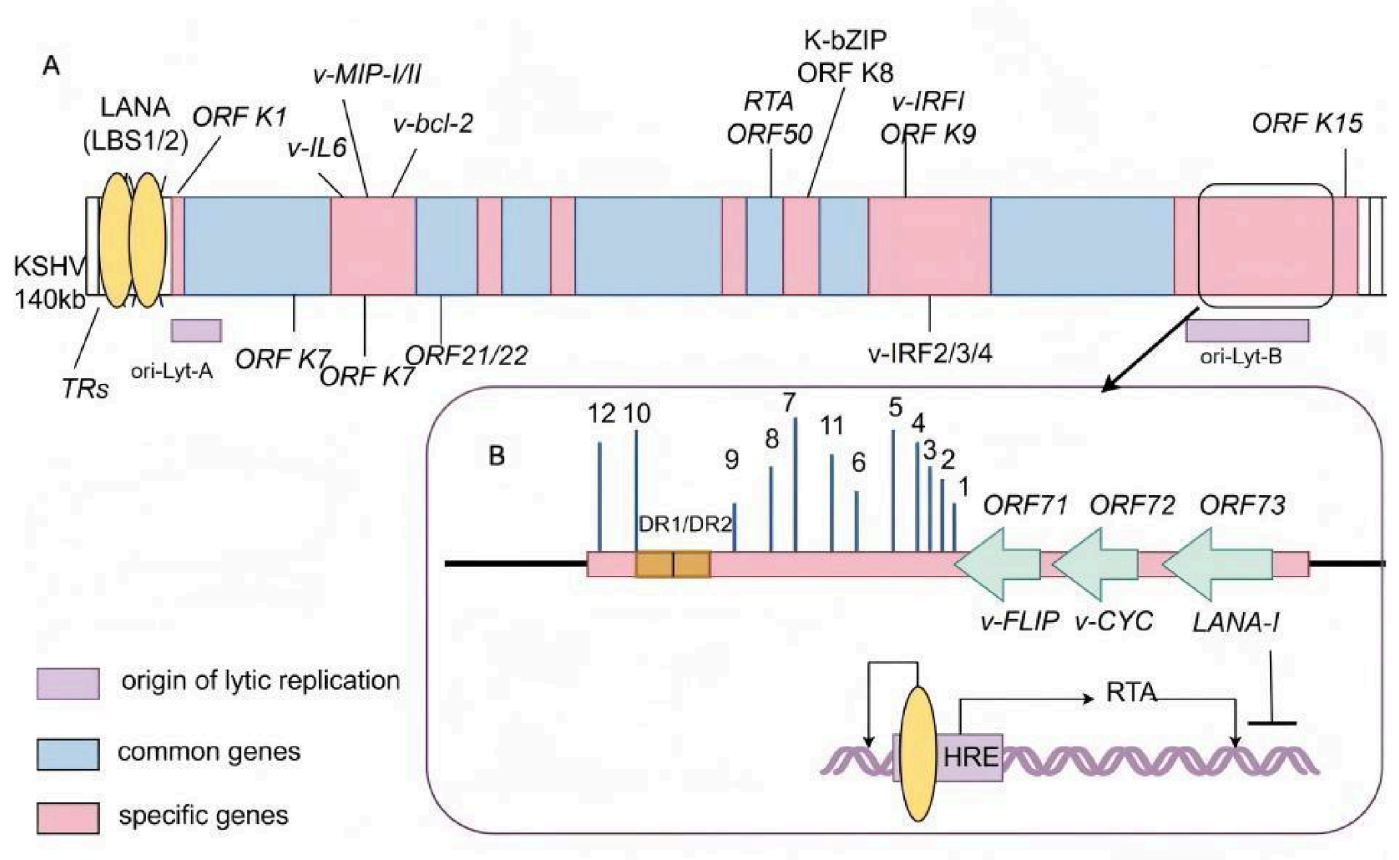
(**A**) The genome of Kaposi’s sarcoma-associated herpesvirus (KSHV) is flanked by GC-rich terminal repeats (TRs), which contain two LANA-binding sites. The entire genome encompasses 90 open reading frames (ORFs). Most ORFs are conserved among human herpesviruses (blue regions), while 15 are KSHV-specific (K1 to K15, pink regions). (**B**) The major latency locus of KSHV is shown in an expanded view. It consists of three core ORFs: ORF73/LANA, ORF72/v-CYC, and ORF71/v-FLIP (indicated by green arrows), with 12 pre-miRNAs represented by blue vertical lines. KSHV-encoded proteins such as LANA and RTA coordinately regulate the switch from viral latency to lytic replication by acting on the RTA promoter.

**Figure 2:**
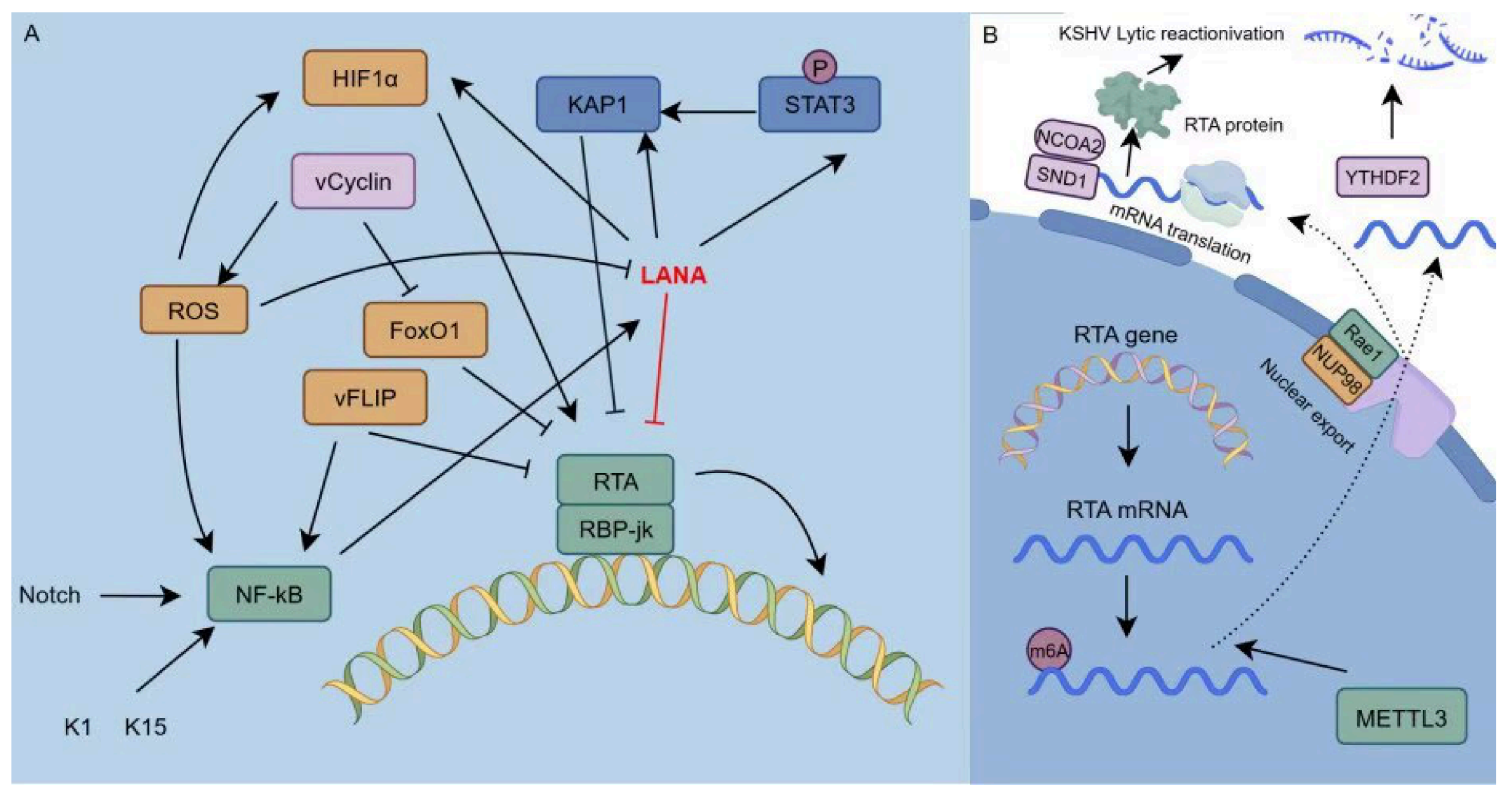
Regulatory mechanisms of KSHV latent maintenance and lytic reactivation. (**A**) The latent nuclear antigen LANA sustains viral latency via a multi-molecular network: it directly represses the transcriptional activity of the RTA-RBP-jκ complex to block the lytic gene expression program; concurrently, it modulates host pathways including HIF1α, STAT3-KAP1 and FoxO1, and acts in concert with viral proteins vCyclin and vFLIP to regulate ROS levels and NF-κB signaling, thereby mediating infected cell survival and proliferation. The Notch pathway and viral proteins K1 and K15 activate NF-κB signaling and are involved in the regulation of latent infection. (**B**) Post-transcriptional regulatory mechanism underlying KSHV lytic reactivation: mRNA transcribed from the RTA gene undergoes m^6^A modification catalyzed by METTL3, followed by nuclear export through the Rae1-NUP98 complex. RTA protein is translated under the regulation of factors such as NCOA2 and SND1, with the m^6^A reader protein YTHDF2 participating in this regulatory process, which ultimately drives KSHV lytic reactivation. In this figure, solid arrows indicate activation or positive regulation, blunt-ended (flat-headed) arrows indicate inhibition or negative regulation, and dashed arrows indicate indirect regulatory relationships or transcriptional/translational processes.

**Figure 3:**
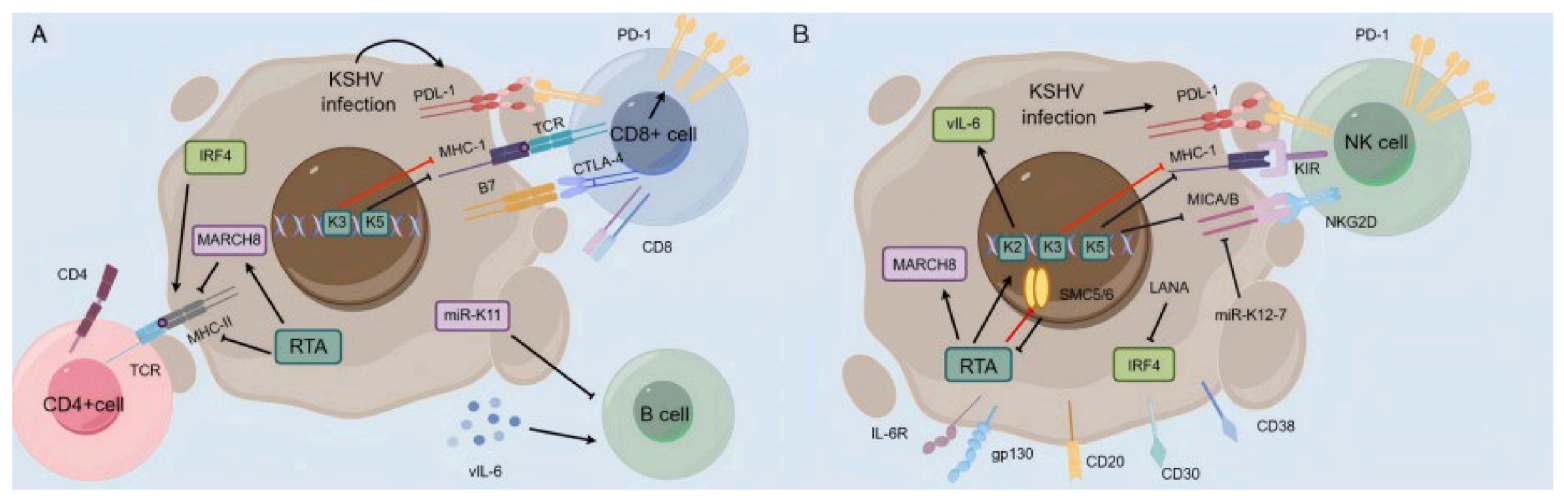
Schematic diagram of KSHV immune evasion mechanisms. (**A**) KSHV-infected cells downregulate MHC-I via viral proteins K3 and K5 to impair antigen recognition by CD8^+^ T cells; they upregulate PD-L1 and act through the B7-CTLA-4 axis to jointly suppress CD8^+^ T cell activation. Viral RTA coordinately regulates MHC-II with host factors IRF4 and MARCH8, thereby modulating CD4^+^ T cell activation. KSHV also regulates B cell functions through vIL-6 secretion and miR-K11 encoding. (**B**) KSHV downregulates MHC-I via K3 and K5 to abrogate inhibitory signals from NK cell KIR receptors, and simultaneously downregulates MICA/B to block NKG2D-mediated NK cell activation, thus achieving innate immune evasion. LANA, IRF4 and miR-K12-7 are involved in the regulation of PD-L1 and NK-activating ligands. vIL-6 promotes cell survival via the IL-6R/gp130 pathway, and RTA antagonizes the SMC5/6 complex to facilitate viral replication. In this figure, solid arrows indicate activation or positive regulation, blunt-ended arrows indicate inhibition or negative regulation.

**Figure 4:**
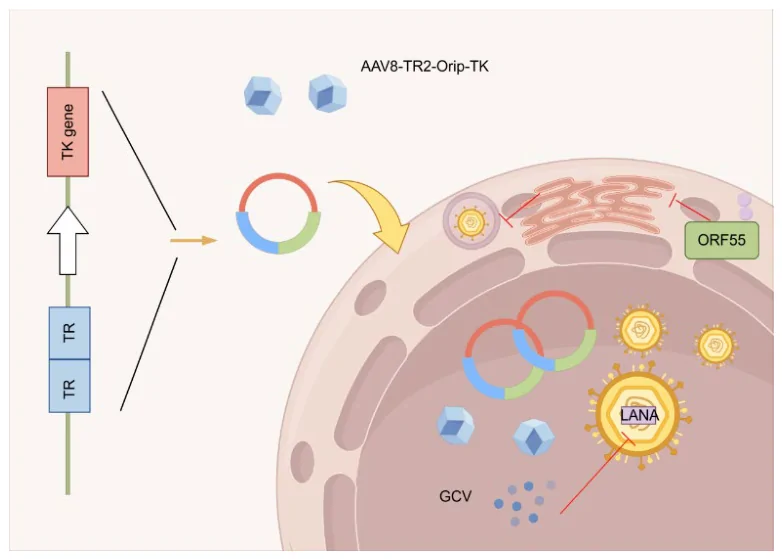
Intervention strategies for key regulatory nodes in KSHV infected cell lysis and viral particle assembly/release. The key intervention strategies include nanoparticle-mediated co-delivery of ganciclovir (GCV) and miR-34a-5p (e.g., ZIF-8-based and microneedle systems), AAV8-TR2-Orip-TK/GCV targeted gene therapy, and inhibition of ORF55 palmitoylation to block Golgi localization and progeny virion assembly. In this figure, solid arrows indicate activation or positive regulation, blunt-ended arrows indicate inhibition or negative regulation.

## Data Availability

This is a clinical medical review. Due to the lack of creation or analysis of new data in this study, data availability is not applicable to this article.
